# EFL Learners' Perceptions of Classroom Justice: Does Teacher Immediacy and Credibility Matter?

**DOI:** 10.3389/fpsyg.2022.925441

**Published:** 2022-06-28

**Authors:** Ruiyun Sun

**Affiliations:** College of Foreign Languages, Northeast Forestry University, Harbin, China

**Keywords:** teacher credibility, teacher immediacy, classroom justice, learners' perceptions, EFL learners

## Abstract

Because learners' perceptions of classroom justice are highly influential on their academic performance, recognizing personal and interpersonal factors that may modify these perceptions seems necessary. Notwithstanding this necessity, a scant number of inquiries have focused on the role of interpersonal factors such as credibility and immediacy in learners' perceptions of classroom justice. In fact, the function of these factors has been overlooked by previous studies. Furthermore, no theoretical review has been performed in this area. To make a stride toward narrowing this lacuna, this theoretical review intends to clarify the role of two interpersonal factors, namely immediacy and credibility, in EFL learners' perceptions of classroom justice. To accomplish this, the previous inquiries were meticulously reviewed. The findings of the review disclosed that EFL learners' perceptions of classroom justice can be considerably influenced by teacher immediacy and credibility. The findings' implications are further discussed.

## Introduction

Classroom justice has always been among the central values of any educational system (Alexander et al., 2021). It is due to the fact that learners' perceptions of classroom justice are proved to be directly associated with their learning outcomes (Burns and DiPaola, 2013; Molinari et al., 2013; Vallade et al., 2014). Classroom justice, also known as classroom fairness, generally pertains to “learners' perceptions of fairness regarding outcomes or processes that occur in the instructional-learning contexts” (Paulsel and Chory, 2005, p. 284). Simply put, it deals with the degree to which classroom outcomes, processes, and interactions are fair in the eyes of learners (Chory et al., 2017). As Estaji and Zhaleh (2021a) articulated, the duty of maintaining justice in classroom settings falls primarily on the teachers' shoulders. To put it another way, teachers, as the main source of power in classroom contexts, are responsible for guaranteeing classroom justice (Sabbagh and Resh, 2014). In this regard, Mameli et al. (2018) also noted that enacting the core principles of justice and fairness in the learning environment is among the main responsibilities of teachers. Teachers, according to Mameli et al. (2018), should behave in a way that their learners perceive as fair. Accordingly, identifying which teacher behaviors learners find fair seems to be crucial. Notwithstanding, a few empirical and review studies in language education have been dedicated to this issue (Kerssen-Griep and Witt, 2012; Argon and Kepekcioglu, 2016; Yan, 2021; Yang, 2021). That is, which teacher personal and interpersonal behaviors may positively influence language learners' perceptions of classroom justice is an open question. To make a stride toward narrowing this gap, this review study intends to delineate the role of two teacher interpersonal behaviors (i.e., immediacy and credibility) using the available evidence.

Teacher immediacy generally pertains to verbal and non-verbal communication behaviors used by instructors to instill feelings of closeness, intimacy, and belonging in their learners (King and Witt, 2009; Velez and Cano, 2012). As noted by Sanders and Wiseman (1990), teacher immediacy includes two underlying aspects of “*verbal immediacy*” and “*non-verbal immediacy*.” The first aspect of teacher immediacy, verbal immediacy, involves verbal communicative actions including “calling students by names,” “asking for students' feedback about the lessons,” “referring to the class as we and our,” and “engaging in conversations with students before and after class” (Liu, 2021, p. 2). Non-verbal immediacy, on the other hand, encompasses non-verbal communicative actions such as “smiling,” “eye contact,” and “direct body orientation” (Nayernia et al., 2020, p. 3). As put forward by Zheng (2021a), using verbal and non-verbal communicative actions enables instructors to promote their learners' motivation and keep them engaged in the mechanism of learning. In this respect, Derakhshan et al. (2022) also maintained that employing immediate communication behaviors, whether verbal or non-verbal, empowers teachers to reduce their students' sense of burnout and disengagement.

Teacher credibility is generally concerned with how firmly learners believe in their instructors (Frymier and Thompson, 1992; Thweatt and McCroskey, 1998). In light of Aristotle's theory of rhetoric, McCroskey (1998) conceptualized this concept as “the attitude of a receiver which references the degree to which a source is seen to be believable” (as cited in Pishghadam and Karami, 2017, p. 380). Subsequently, Myers and Martin (2018) defined teacher credibility in terms of its underlying components (i.e., competence, caring, and character). Teacher credibility, according to Myers and Martin (2018), relates to the degree to which students perceive their teachers to be knowledgeable, responsive, and trustworthy. As Derakhshan (2021) mentioned, being perceived as credible paves the way for teachers to engage learners in learning activities. Pishghadam et al. (2017) also noted that perceptions that learners make about the credibility of their instructors are highly influential in their learning outcomes. Further, Pishghadam et al. (2021) posited that those students who have a positive impression of their instructors' character are more willing to communicate in the classroom environment.

Because of the significance of teacher immediacy and credibility (Pishghadam et al., 2017; Derakhshan et al., 2022), a substantial number of investigations have been devoted to these constructs. A series of studies have explored the impact of teachers' immediacy and credibility on teachers themselves and their professional success (e.g., Nayernia et al., 2020). The rest have focused on the effects of these two constructs on learners and their academic behaviors, including engagement (e.g., Dixson et al., 2017; Stilwell, 2018; Zheng, 2021b), willingness to communicate (e.g., Yu, 2011; Fallah, 2014; Pishghadam et al., 2019; Sheybani, 2019; Lee, 2020), motivation (e.g., Estepp and Roberts, 2015; Furlich, 2016; Frymier et al., 2019; Karimi and Ziaabadi, 2019; Megawati and Hartono, 2020; Hussain et al., 2021), burnout (e.g., Gholamrezaee and Ghanizadeh, 2018; Derakhshan et al., 2022), and academic achievement (e.g., Mazer and Stowe, 2016; Kalat et al., 2018; Violanti et al., 2018; Ge et al., 2019). Nonetheless, the potential effects of these communication behaviors on learners' perceptions of classroom justice have remained elusive. Simply put, limited empirical investigations have been performed in this respect (Kerssen-Griep and Witt, 2012; Argon and Kepekcioglu, 2016; Yan, 2021; Yang, 2021). Further, to the authors' knowledge, no review study has delved into the consequences of teacher immediacy and credibility for learners' perceptions of classroom justice. To address the gap, the present review study strives to illustrate the role of teachers' immediacy and credibility in EFL learners' perceptions of classroom justice.

### Teacher Immediacy

The notion of immediacy generally pertains to the emotional, psychological, and physical proximity that exists between communicators (Mehrabian, 1969, 1971; Velez and Cano, 2008, 2012). Teacher immediacy, according to LeFebvre and Allen (2014), refers to the sense of intimacy and closeness that learners experience as a result of their teachers' verbal and non-verbal behaviors. Verbal behaviors include but are not limited to the following: “addressing learners by their first names”, “asking for learners' feedback about the lessons”, and “referring to the class as we and our” (Roberts and Friedman, 2013). On the other hand, non-verbal behaviors involve “eye contact”, “body posture”, “smiling”, and “head nods” (York, 2013). As Hsu (2010) mentioned, the concept of teacher immediacy has its roots in approach-avoidance theory. This theory suggests that “people commonly approach what they like and avoid what they don't like” (Wang and Schrodt, 2010, p. 28). Extending this assumption to the educational domain, learners approach teachers they like and avoid those they do not (Seifu and Gebru, 2012). Building upon the approach-avoidance theory, Estepp and Roberts (2015) argued that learners are typically attracted to those teachers who strive to decrease the emotional and psychological distance using verbal and non-verbal cues.

### Teacher Credibility

Credibility, in a broad sense, deals with the audience's perceptions regarding the believability of the speaker (McCroskey et al., 2006; Bolkan and Goodboy, 2009). Likewise, teacher credibility relates to the extent to which pupils have faith in their teachers (Zhang, 2009). This construct is rooted in Aristotle's theory of rhetoric, which suggests that credibility as the most efficient means of persuasion comprises three major dimensions, including “*intelligence*,” “*character*,” and “*goodwill*” (Santilli et al., 2011). Intelligence, or perceived knowledge, pertains to “teachers' expertise in the subject that (s)he is teaching” (Derakhshan, 2021, p. 8). Character also refers to the teachers' degree of trustworthiness (Ledbetter and Finn, 2018). Finally, goodwill, or caring, relates to “the degree of teachers' attention to students' feelings, desires, and interests” (Won et al., 2017, p. 534). Taken as a whole, teacher credibility is concerned with how firmly learners believe in their teachers' character, intelligence, and goodwill. As Xie and Derakhshan (2021) noted, a knowledgeable, caring, and trustworthy teacher who is reliable in the eyes of his/her learners can readily persuade them to attend classes and take part in the learning process.

### Classroom Justice

Classroom justice generally refers to the individual learners' judgments of the fairness of processes, procedures, and outcomes (Chory, 2002; Horan and Myers, 2009). As a multi-dimensional concept, classroom justice comprises three fundamental facets, including “*distributive justice*,” “*interactional justice*,” and “*procedural justice*” (Horan et al., 2013; Chory et al., 2014). Distributive justice, as the first facet, pertains to “the perception that the distribution of grades is fair” (Horan et al., 2010, p. 455). The principles of equity, need, and equality are used to realize this facet (Jasso et al., 2016). This facet is thought to be maintained when learners perceive that the distribution of outcomes is based on their academic endeavors, contributions, and performance (Cropanzano et al., 2015; Ehrhardt et al., 2016; Estaji and Zhaleh, 2022). The second facet of classroom justice, interactional justice, refers to “to perceptions of fairness in the interpersonal treatment received by individuals, mainly as far as the communicative and relational requests that learners address to their teachers are concerned” (Berti et al., 2010, p. 543). This facet is believed to be sustained when learners feel they are in a respectful atmosphere where they are treated politely and information is conveyed to them in a regular, honest, and rational manner (Kazemi et al., 2015; Estaji and Zhaleh, 2021b). As the last facet, procedural justice refers to learners' impressions of the mechanisms employed to allocate outcomes (Claus et al., 2012). This facet is deemed to be preserved when the methods are considered to be unbiased, formed on true and adequate information, used consistently across time and individuals, adjustable, and implemented truthfully (Kazemi, 2016; Rasooli et al., 2019).

### The Role of Teacher Credibility and Immediacy in EFL Learners' Perceptions of Classroom Justice

Concerning the role of teacher credibility in EFL learners' perceptions of classroom justice, Paulsel et al. (2005) proposed that a credible instructor is capable of enacting and maintaining distributive justice in instructional-learning contexts. They argued that individual learners who believe in their teachers' competence also trust them to assess their academic performance competently. To them, having faith in teachers' knowledge leads language learners to perceive that the scores assigned by their teachers are fair. As such, EFL learners' perceptions of distributive justice appear to be favorably influenced by their positive impressions of teachers' knowledge. In this respect, Chory (2007) also stated that reliable instructors can efficiently implement the main assumptions of procedural justice as well. She suggested that “language learners may generalize their perceptions of the instructor's expertise in the subject matter to the instructor's knowledge regarding the best procedures to use in teaching that subject” (p. 94). Accordingly, EFL learners' perceptions of procedural justice seem to be positively affected by their favorable viewpoints regarding teachers' competence. In a similar vein, Lankiewicz (2014) posited that credible teachers are successful at preserving interactional justice in classroom contexts. It is due to the fact that credible teachers are not only competent in teaching the subject matter but also expert in interacting with learners (Schrodt and Finn, 2011; Witt et al., 2014). Based on this, competent and knowledgeable teachers can significantly improve EFL learners' perceptions of interactional justice. Besides, regarding the role of teacher immediacy in EFL learners' perceptions of classroom justice, Kerssen-Griep and Witt (2012) asserted that teachers' verbal and non-verbal cues significantly predict EFL learners' fairness perceptions. They explained that immediate teachers commonly provide learners with an intimate and respectful instructional-learning atmosphere wherein the central assumptions of interactional justice can be realized.

## Empirical Evidence

To date, a few investigations have been conducted into the role of teacher immediacy and credibility in learners' perceptions of classroom justice (Kerssen-Griep and Witt, 2012; Argon and Kepekcioglu, 2016; Yan, 2021; Yang, 2021). In their study, Kerssen-Griep and Witt (2012) examined the extent to which learners' fairness perceptions can be predicted by teachers' immediacy behaviors. To accomplish this, the valid measures of the constructs were given to 269 university students. Inspecting the correlation of scales, the researchers found a direct relationship between teacher immediacy and learners' fairness perceptions. Using regression analysis, they also discovered that teachers' verbal and non-verbal immediacy are powerful predictors of fairness perceptions. In another study, Argon and Kepekcioglu (2016) inspected the association between teacher credibility and learners' perceptions of classroom justice. To do so, 1,439 university students were selected to respond to two close-ended scales, namely “*Justice in the Classroom*” and “*Instructor Credibility*.” The correlational analysis outcomes demonstrated a favorable association between teacher credibility and learners' perceptions of classroom justice. By the same token, Yang (2021) assessed the function of teachers' positive behaviors in pupils' perceptions of distributive, procedural, and interactional justice. The findings demonstrated that teachers' positive behaviors, including immediacy and credibility, can favorably influence students' perceptions of justice. More recently, Yan (2021) evaluated the impact of teacher immediacy on EFL learners' perceptions of classroom justice. To do this, a series of pre-designed scales were distributed among 1,178 EFL learners. Implementing SEM analyses, the researchers discovered that teachers' verbal and non-verbal behaviors can modify learners' perceptions of classroom justice.

## Conclusion and Pedagogical Implications

In this theoretical review, the constructs of teacher credibility, teacher immediacy, and classroom justice were thoroughly characterized. Moreover, the previous investigations performed on these constructs and their relationships were fully reviewed. Finally, the existing premises regarding the impacts of teacher credibility and immediacy on learners' perceptions of justice were briefly explained. With respect to the empirical and theoretical evidence, one can logically infer that both credibility and immediacy can modify EFL learners' perceptions of classroom justice. Simply put, EFL learners' impressions of distributive, interactional, and procedural justice can be remarkably improved by teachers' credibility and immediacy. This finding appears to be instructive for all teachers, instructors, and educators in any instructional-learning context, particularly English language classes. Taking the findings of this review into account, teachers need to employ verbal and non-verbal communicative actions to enact the assumptions of interactional justice in classrooms. They are also required to improve their instructional skills and subject knowledge because being perceived as credible empowers them to greatly influence learners' perceptions of procedural and distributive justice. The outcome of this theoretical review also seems to be insightful for teacher educators. Given the important role verbal and non-verbal behaviors serve in modifying learners' perceptions of fairness, teacher educators should teach their teacher students how to use immediate behaviors in instructional-learning environments. Besides, owing to the paucity of research on the effects of teacher communication behaviors on learners' fairness perceptions, future investigations into this topic are highly advised.

## Author Contributions

RS independently conceptualized and drafted the current study. Then the author approved its current manuscript to Front. Psycho.

## Funding

This study was supported by the Fundamental Research Funds for the Central Universities-Studies on Discourse Analysis and Language Cognition from an Interdisciplinary Perspective (Grant No. 2572021DF07) and Teaching Reform Project of Higher Education in Heilongjiang Province-Research on English Teaching Strategies based on Critical Thinking Ability (Grant No. SJGY20190037).

## Conflict of Interest

The author declares that the research was conducted in the absence of any commercial or financial relationships that could be construed as a potential conflict of interest.

## Publisher's Note

All claims expressed in this article are solely those of the authors and do not necessarily represent those of their affiliated organizations, or those of the publisher, the editors and the reviewers. Any product that may be evaluated in this article, or claim that may be made by its manufacturer, is not guaranteed or endorsed by the publisher.
